# Mannitol for cerebral oedema after acute intracerebral haemorrhage (MACE-ICH): protocol for a prospective, randomised, open-label, blinded-endpoint phase IIb trial

**DOI:** 10.1136/bmjopen-2025-103776

**Published:** 2025-07-28

**Authors:** Kailash Krishnan, Emma Grace, Lisa Woodhouse, Christine Roffe, Jesse Dawson, Timothy J England, David W Hewson, Rob A Dineen, Zhe Kang Law, Stefan Pszczolkowski, Keenan Wells, Amanda Buck, Jennifer Craig, Diane Havard, Mary Joan Macleod, David J Werring, Fergus Doubal, Nikola Sprigg, Philip Bath

**Affiliations:** 1 University of Nottingham, Nottingham, UK; 2 Department of Acute Medicine, Nottingham University Hospitals NHS Trust, Nottingham, UK; 3 Stroke Trials Unit, Mental Health & Clinical Neurosciences, University of Nottingham, Nottingham, UK; 4 Institute for Science and Technology in Medicine, Keele University, Keele, UK; 5 Institute of Cardiovascular and Medical Sciences, University of Glasgow, Glasgow, UK; 6 University of Nottingham, Derby, UK; 7 Stroke Medicine, Royal Derby Hospital, Derby, England, UK; 8 Nottingham University Hospitals NHS Trust, Nottingham, UK; 9 Radiological Sciences, Academic Unit of Mental Health and Clinical Neuroscience, University of Nottingham, Nottingham, UK; 10 NIHR Nottingham Biomedical Research Centre, Nottingham, UK; 11 Radiology, Queen’s Medical Centre, Nottingham, UK; 12 Department of Medicine, National University of Malaysia, Kuala Lumpur, Malaysia; 13 Radiology Department, Nottingham University Hospitals NHS Trust, Nottingham, UK; 14 Mental Health & Clinical Neuroscience, University of Nottingham, Nottingham, UK; 15 6Division of Clinical Neuroscience, University of Nottingham, Nottingham, UK; 16 University of Aberdeen Division of Applied Medicine, Aberdeen, UK; 17 UCL Institute of Neurology, London, UK; 18 Department of Brain Repair and Rehabilitation, University College London Stroke Research Centre, London, UK; 19 The University of Edinburgh, Edinburgh, UK

**Keywords:** Clinical trials, Intracerebral Hemorrhage, STROKE MEDICINE

## Abstract

**Background:**

Acute intracerebral haemorrhage (ICH) is devastating with a 1 month mortality rate of ~40%. Cerebral oedema can complicate acute ICH and is associated with poor outcome. In patients with large ICH, the accompanying swelling increases mass effect and causes brain herniation. Mannitol, an osmotic diuretic, is used to treat cerebral oedema after traumatic brain injury, but its safety and efficacy in ICH is unclear. We aim to assess the feasibility of a phase II randomised, controlled trial of mannitol in patients with ICH with, or at risk of, cerebral oedema to inform a definitive trial.

**Methods:**

The mannitol for cerebral oedema after acute intracerebral haemorrhage trial (MACE-ICH) aims to include 45 ICH participants from 10 UK sites with estimated largest diameter of haematoma volume >2 cm, presenting within 72 hours of onset with, or at risk of, cerebral oedema (limited Glasgow Coma Scale (GCS)<9, including motor and visual components only, and National Institutes of Health Stroke Scale>8) with or without mass effect. Participants will be randomised (1:1:1) to 1 g/kg 10% single-dose intravenous mannitol, 1 g/kg 10% mannitol followed by a second dose at 24 hours, or standard care alone. Outcome assessors will be masked to treatment allocation. Feasibility outcomes include proportion of patients approached being randomised, participants receiving allocated treatment, recruitment rate, treatment adherence and follow-up. Secondary outcomes include serum electrolytes and osmolality at days 1–2; change in ICH and oedema volume at day 5; number of participants who developed urinary tract infection, GCS and National Institutes of Health Stroke Scale at day 5±2; length of hospital stay, discharge destination and death up to day 28; death and death or dependency by day 180 and disability (Barthel Index), quality of life (EuroQol, 5-D) and cognition (telephone mini-mental state examination) at day 180.

**Ethics and dissemination:**

MACE-ICH received ethics approval from the East Midlands-Leicester Central research ethics committee (22/EM/0242). The trial is funded by a National Institute for Health and Care Research RfPB grant (203080). The results will be published in an academic journal and disseminated through academic conferences and patient support groups. Reporting will be in line with Consolidated Standards of Reporting Trials recommendations.

**Trial registration numbers:**

ISRCTN15383301; EUDRACT 2022-000283-22.

**STRENGTHS AND LIMITATIONS OF THIS STUDY**

- Acute intracerebral haemorrhage (ICH) is a devastating stroke for which treatment lags significantly when compared with ischaemic stroke. Mannitol for cerebral oedema after acute intracerebral haemorrhage trial (MACE-ICH) aims to test mannitol, which is an osmotic diuretic, as a potential treatment for cerebral oedema after ICH.
- This is a feasibility trial with candidate clinical and radiological outcomes to determine if a definite trial is feasible; MACE-ICH is the first trial which will compare two doses of mannitol versus standard care alone.
- Recruitment into MACE-ICH is based on routine clinical assessment and CT scanning, which is quick, widely available and an inexpensive imaging resource.
- Local investigators and clinical staff administering mannitol are not blinded, but follow-up assessments and adjudication of brain imaging will be performed by assessors blinded to treatment allocation.

## Introduction

 Acute spontaneous intracerebral haemorrhage (ICH) accounts for up to 15% of 150 000 strokes each year in the UK, and to date, management is largely supportive.^1 2^ Mortality in ICH is significantly higher compared with ischaemic stroke (~34% vs 12% at 1 month), and data from a UK-based registry indicate that up to 80% of survivors are left with significant disability long term.^3^ The clinical spectrum and prognosis vary according to age, intraventricular extension of haematoma and location, with more severity and mortality associated with lobar haematoma compared with deep subcortical location.^4 5^ One potential explanation is that non-hypertensive mechanisms such as cerebral amyloid angiopathy lead to lobar ICH, while hypertension is more associated with deep ICH.^4^ Aside from lowering blood pressure, reversal of coagulopathy and surgical evacuation of haematoma in a proportion of patients and management of ICH remains largely supportive.^1 6^ Consequently, research into complications such as cerebral oedema is warranted. Oedema starts within hours of ICH from clot retraction releasing serum into the surrounding brain, peaks at around 72 hours and continues for days following blood-brain barrier disruption and inflammation.^7^,^9^ Large ICH (~15%) causes significant swelling,^10^ and the only treatment supported by evidence is surgical decompression.^2^ However, surgery is not routinely available, and older patients with comorbidities, who comprise the majority of haemorrhagic strokes, are often ineligible. Furthermore, surgery is associated with risks and major complications.

Mannitol, an osmotic diuretic, is readily available in most UK hospitals and easy to administer intravenously. It is licensed to treat cerebral oedema and used in traumatic brain injury and hepatic encephalopathy.^11 12^ Mannitol is known to stroke physicians: some use it regularly, occasionally or never.^13^ It is known to lower intracranial pressure by drawing water from the brain interstitium into the intravascular space.^11 14^ Mannitol is also thought to reduce blood viscosity, facilitating blood flow and oxygen delivery to the brain.^9 15^ As a free radical scavenger, mannitol might act as a neuroprotectant.^9 16^ Little is known about its effects in ICH: to date, studies have been low quality and underpowered.^1317^,^19^ Guidelines do not recommend the routine use of mannitol,^1 20^ so well-designed, randomised controlled trials (RCTs) in ICH are urgently needed.^21^

## Methods

### Aim

The mannitol for cerebral oedema after acute intracerebral haemorrhage trial (MACE-ICH) aims to determine the feasibility and inform the design and conduct of an adequately powered, pragmatic prospective multicentre RCT.

### Trial design

MACE-ICH is a multicentre, UK-based, three-arm, prospective, randomised, open-label, blinded-endpoint feasibility trial. The overarching hypothesis is that mannitol will reduce cerebral oedema after ICH and thereby improve outcome. This trial is funded by the National Institute for Health and Care Research, Research for Patient Benefit Programme, and is registered with a UK-based registry (ISRCTN15383301). MACE-ICH started recruiting participants in February 2024, and the trial is scheduled to be closed in November 2025.

### Eligibility criteria

#### Inclusion

- Adults (>18 years).
- Spontaneous ICH confirmed by CT scan with an estimated largest diameter of haematoma>2 cm.
- <72 hours of ictus (or from last seen healthy).
- Cerebral oedema with or without evidence of mass effect.
- At risk of cerebral oedema (limited Glasgow Coma Scale (GCS) score<9 (eye opening and motor component only) and National Institutes of Health Stroke Score (NIHSS)>8).
- Signed consent (patient, personal or professional representative or independent physician).

In the event that more than one scan is available for a particular patient, the investigator will assess eligibility taking into account the clinical information and the latest scan which best meets the inclusion criteria. The scan used for eligibility will be documented and collected. This pre-enrolment scan will be used to compare with the post-treatment scan at day 5±2.

#### Assessment of maximum haemorrhage diameter and cerebral oedema for eligibility

Visual estimate of maximum haematoma diameter (>2 cm) and presence of cerebral oedema will be performed by investigators at sites prior to enrolment by reviewing the plain CT scan viewed at the local hospital. Maximum haematoma diameter is assessed as the longest haematoma diameter in any plane, based on a measurement made with the calliper tool in PACS, Picture Archiving and Communication System (if available), or by visual comparison to the image measurement scale embedded in the image. Cerebral oedema is identified as a hypodense zone surrounding the haematoma.^22^

#### Exclusion

- GCS<5.
- Premorbid modified Rankin Scale (mRS)>3.
- Isolated subarachnoid haemorrhage.
- Haemorrhage known to be from trauma, venous thrombosis, arteriovenous malformation, brain tumour, transformation of cerebral infarct, cerebral aneurysm or thrombolytic drug.
- Known hypersensitivity to mannitol.
- Severe renal failure (eGFR (estimated Glomerular Filtration Rate)<30 mL/min or dialysis).
- Cardiac failure.
- Hypotension at baseline (SBP (Systolic Blood Pressure)<90 mm Hg).
- Anuria.
- Patient unwilling to participate.
- Geographical or other factors which prohibit follow-up.
- Pre-existing comorbidity with preictal life expectancy<6 months.
- Severe dementia.
- Planned for palliative care.
- Severe hypernatremia (sodium>160 mmol/L) and serum osmolality>320 mosmol/kg.
- Severe hyponatraemia (sodium<125 mmol/L).
- Women of childbearing potential with a positive pregnancy test at the time of admission or lactating.
- Patients in whom peripheral intravenous cannula cannot be placed.
- Planned neurosurgery.

### Consent

**Figure 1:**
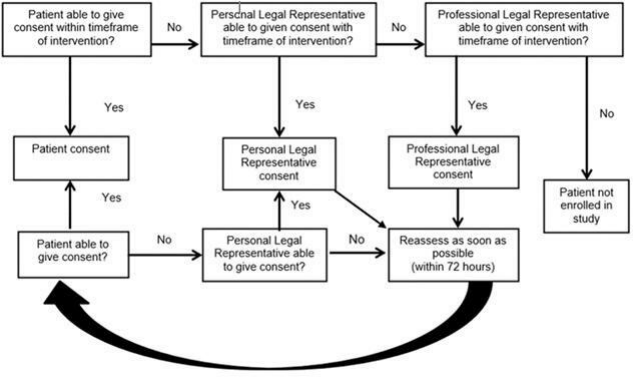
Flowchart for the process of consent.

Patients with the capacity to give consent will be approached directly. If the patient is unable to write (eg, dominant hand weakness, dyspraxia and ataxia), witnessed verbal consent (or a mark made by the participant with intent to sign) may be recorded on the consent form. If a participant lacks the mental capacity to provide consent due to decreased consciousness or aphasia, the investigator or nominee will approach the patient’s relative (or other personal legal representative such as partner or close friend) to seek consent (figure 1). If this is not possible, then an independent medical practitioner not associated with the trial will be approached for proxy consent.^23^ Full informed consent is later sought from the recruited participant within 72 hours if mental capacity is regained (figure 1).

It will be explained to the patient or their legal representative that enrolment into the trial is entirely voluntary and they can withdraw at any time without giving a reason.

### Randomisation

All participants eligible for inclusion and for whom consent will be obtained will be randomised in real time over a secure internet site with minimisation on prognostic factors including age, time since stroke onset, limited GCS and visual estimate of maximum haematoma diameter.

In the event that the trial website cannot be accessed or server failure occurs, investigators will follow the working practice document for disaster recovery, which will allow the participant to be randomised following a standard operating procedure.

The unpredictable allocation sequence will be generated and programmed into the computerised randomisation system. The research team at each site will conduct the randomisation via secure log-ins to the web-based system. Randomisation using a minimisation algorithm (age<70 years or older, time to randomisation<24 hours, limited GCS score 2–6, ICH diameter 4 cm or greater) and release of allocation only after enrolment, consent and baseline data collection will ensure allocation concealment.

This trial will compare two doses of mannitol versus standard care including monitoring of participants before, during and after treatment, collection of key clinical assessments and presence or absence of adverse effects. It is not feasible for participants and researchers/investigators to be blinded due to the open-label design. Clinical staff preparing and administering mannitol will also not be blinded to treatment allocation. However, follow-up assessments and adjudication of brain imaging will be conducted centrally by assessors blinded to randomisation and treatment allocation.

As this is an unblinded trial, code-breaking will not be required. If a contra-indication to mannitol develops after randomisation (eg, anuria or severe congestive cardiac failure), the trial treatment should be stopped. In order to minimise bias that could be introduced through knowledge of which treatment the participant has received, unblinded staff will be kept to a minimum and will be asked not to reveal treatment allocation to anyone.

### Intervention

**Figure 2:**
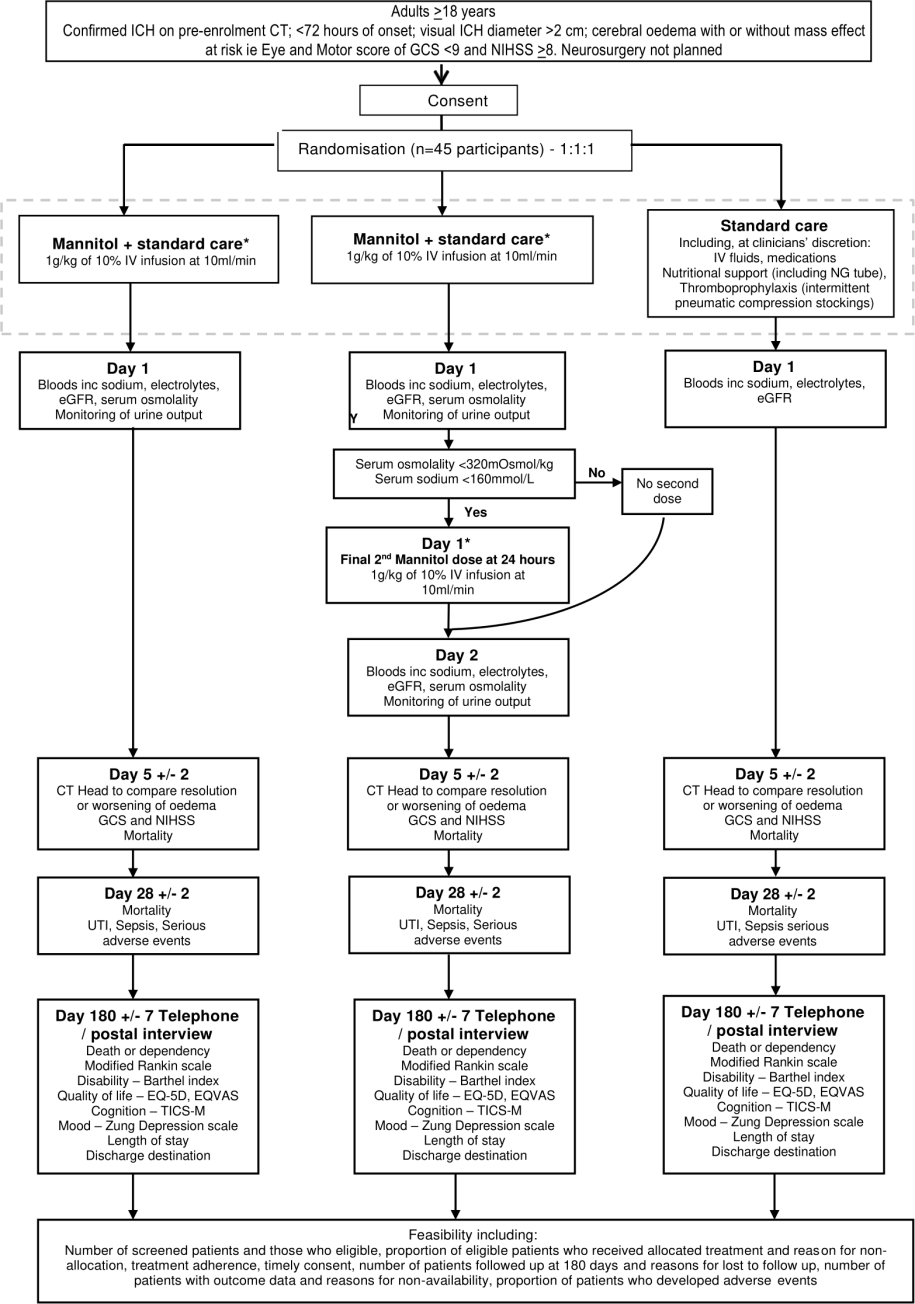
Trial flowchart. ICH, intracerebral haemorrhage; IV, intravenous; GCS, Glasgow Coma Scale; NIHSS, National Institutes of Health Stroke Score; TICS-M, Telephone Interview of Cognitive Status; NG, nasogastric; e-GFR, estimated Glomerular Filtration Rate; UTI, Urinary Tract Infection; EQ-5D, European Quality of Life 5 Dimensions score; EQ-VAS, EuroQoL Visual Analogue Scale.

Participants will be enrolled equally into one of three groups (figure 2) as soon as possible after randomisation:

- Arm 1: 1 g/kg 10% single-dose mannitol infusion at 10 mL/min, in addition to standard care.
- Arm 2: 1 g/kg 10% mannitol at 10 mL/min followed by a second dose 1 g/kg repeated at 24 hours, if the serum osmolality is <320 mOsm/kg and sodium<160 mmol/L after the first dose, in addition to standard care.
- Arm 3: standard care alone.

### Assessments

Participants will be assessed clinically at baseline (demographics, medical history, investigations, diagnosis of ICH) in line with local clinical practice and preinfusion (day 1) and end of treatment (day 1–2) for Arms 1 and 2. All participants will be assessed again on day 5 (±2), hospital discharge and on day 180 to gather information on interventions, adverse events, discharge data and discharge destination. Data collection forms can be found in online (https://stroke.nottingham.ac.uk/mace-ich/docs/).

All participants will have brain imaging (CT head) as part of routine care before enrolment. A second research CT head will be performed at day 5 (±2) after intervention to measure haemorrhage volume and oedema volume. The methods of scan analysis are detailed in the online supplemental document.

#### Blood tests

All participants will have blood tests taken as part of routine clinical care at the time of presentation whether or not they go on to participate in the trial. Blood samples will be labelled and analysed locally at each hospital site in accordance with local practice. Each participant in Arms 1 and 2 will have one blood test for urea and electrolytes (U&Es) and serum osmolality after the infusion, and the results will be made available to the treating clinical team. Participants in Arm 2 will have a further blood test after the second infusion (figure 2).

### Outcomes

The primary outcome is based on feasibility: number of patients screened, rate of eligible patients randomised and reasons for non-randomisation, participants who received allocated treatment and adherence to intervention, proportion of participants followed up to day 180 and reasons for loss to follow-up, proportion of randomised participants with full outcome data and reasons for non-availability.

The following measures are expected to be secondary outcomes in a definitive trial and will be collected:

- Day 1–2: U&Es; eGFR; serum osmolality to correlate response to mannitol.
- Day 5±2: neurological deterioration (increase in NIHSS>3); GCS; NIHSS; death; urinary tract infection; sepsis.
- Radiological: follow-up CT after treatment (day 5±2) for comparison with pre-enrolment imaging to assess changes in oedema volume, haematoma size/volume, maximum diameter, midline shift and hydrocephalus.
- Day 28: mortality; urinary tract infection; septicaemia.
- Day 180 (blinded central follow-up via telephone/postal interview): death or dependency (mRS) disability (Barthel Index); mood (Zung depression scale); cognition (Telephone Interview of Cognitive Status); quality of life (EuroQol 5-D); length of stay; discharge destination. The long-term outcomes post-COVID-19 and ICH are not yet fully defined and may include additional yet undefined neurological complications. These will also be collected.
- Number of patients transferred to the high dependency unit.
- Number of patients intubated and ventilated.
- Number of patients who underwent neurosurgical intervention.
- Recurrent stroke (ischaemic or haemorrhagic).

### Adverse events

All adverse events on days 0–1 (including during infusion, Arms 1 and 2 only) and for 24 hours post-treatment will be collected. All adverse events will be assessed for seriousness, expectedness and causality by adjudicators masked to treatment allocation. Serious adverse events (SAEs) will be categorised in accordance with the medical dictionary for regulatory authorities. All SAEs will be collected until day 28, and fatal SAEs and safety outcome events (thrombophlebitis, hyper/hyponatraemia, pulmonary oedema, hypotension, renal impairment) will be collected until day 180.

### Statistical analysis

A medical statistician blinded to treatment allocation will support analysis using a validated software package. A preplanned statistical analysis plan will be confirmed before the database is locked and release of randomisation codes. The trial results will be reported in accordance with the Consolidated Standards of Reporting Trials guidelines.

This is a feasibility study, and the analysis will be using descriptive statistics. Continuous variables will be summarised using means and SD or medians and IQRs, depending on distribution. While some variables will be listed by treatment groups, any analysis will be exploratory.

It is planned for this feasibility study to be undertaken at Nottingham University Hospitals NHS Trust in partnership with nine other acute UK hospitals over 24 months. Lower recruitment would not preclude progression if there was evidence that barriers could be overcome.

### Sample size and justification

Given the primary aim of this trial is feasibility, which includes the objective of assessing the rate of recruitment, a formal sample size calculation is not appropriate. Stroke trials have recruited participants with a similar profile to MACE-ICH, so it is anticipated that recruiting 45 patients with a high rate of compliance and follow-up data would support the basis for a further grant application to undertake a definite trial.

Depending on the final sample size calculation for the definitive study, the number of centres and recruitment period could be determined using the information from the rates and recruitment patterns in the feasibility study. Information about set-up times for hospital sites will inform projections for a larger study.

### Patient and public involvement

This trial was developed in collaboration and supported by the Nottingham Stroke Research Partnership group, comprising stroke survivors and their carers. Members of this group reviewed this proposal and, in an iterative process, commented on the study design and conduct. Broad inclusion criteria were developed to reflect real-life presentation of patients to stroke units, including those with multiple medical problems. In addition, the members also felt that all should be done to ensure that potential participants are not to be denied access to the trial because they have no capacity. The feedback informed the approach of taking informed consent in this study.

###  Ethical approval and dissemination

MACE-ICH received a favourable ethical opinion from the East Midlands-Leicester Central Research Ethics Committee (REC) (22/EM/0242). The trial is being adopted in the UK by the National Institute for Health and Care Research.

The results will be published in a peer-reviewed academic journal and disseminated through academic conferences and stroke patients’ support groups.

### Protocol amendments

Should a protocol amendment be made that requires ethical approval, the changes will not be implemented until the amendment and the revised consent forms, information sheets have been reviewed and approval is received from the REC, Medicines and Healthcare products Regulatory Agency and research and development departments. Minor protocol amendments only for logistical or administrative changes may be implemented immediately, and the REC will be informed. The results will be communicated to all principal investigators.

### Confidentiality and access to data

All trial staff will endeavour to protect the right of the participant’s privacy and informed consent and will adhere to the Data Protection Act 2018. Case report forms will only collect the required information for the trial and store it safely in confidential conditions. Access to the information will be limited to the trial staff and investigators and relevant regulatory authorities. Computer-held data including the trial database will be held securely and password protected. All data will be stored on a secure dedicated web server. Access will be restricted by username identifiers and passwords (encrypted using a one-way encryption method). Information about the trial in the participant’s medical records/hospital notes will be treated confidentially in the same way as other confidential information. Electronic data will be backed up every 24 hours to both local and remote media in encrypted format.

### Insurance and indemnity

Insurance and indemnity for trial participants and trial staff are covered within the National Health Service Indemnity Arrangements for clinical negligence claims within the NHS (National Health Service). There are no special compensation arrangements for this trial, but participants may have recourse through the NHS complaints procedures. Nottingham University Hospitals NHS Trust is the sponsor of this study.

### Trial management

A trial management committee based at the Stroke Trials Unit, Nottingham, UK, is responsible for the day-to-day conduct of the trial. The trial will be overseen by a Trial Steering Committee (TSC). An independent Data Monitoring Committee (DMC) receives safety reports every 6 months or more frequently if requested and performs unblinded reviews of safety data. The DMC reports their assessment to the chair of the TSC. Collaborators and researchers associated with the trial may write through the trial office to the DMC to draw attention to any concern they may have about the trial, interventions or any other relevant issues.

The study data will be collected and analysed by the Stroke Trials Unit at Nottingham. The trial will be conducted in full conformity with the Declaration of Helsinki, 2013, principles of Good Clinical Practice, Medicines for Human Use Regulations and the UK Policy Framework for Health and Social Care Research.

## Discussion

The use of mannitol in ICH is promising and has been the traditional hyperosmolar agent of choice in management of cerebral oedema. MACE-ICH has therefore been designed as an open, pragmatic clinical trial to determine the feasibility of administering mannitol in adult patients with ICH and cerebral oedema or those at significant risk. This trial will inform a definite trial which will compare the safety and efficacy of a single dose of mannitol, two doses compared with standard care. If oedema is prevented or reduced, outcomes may improve.

In this trial, broad inclusion criteria have been developed to reflect the diverse presentation of patients with ICH. The clinical course in the early hours and days after ICH is dynamic, so patients who do not fit the inclusion criteria on admission will be eligible if they subsequently deteriorate and develop signs or radiological features which meet the inclusion criteria within 72 hours. The optimal timing for treatment is unknown, and the rationale for <72 hours is based on when patients are likely to develop brain swelling and/or are at risk of further expansion.^10^ The rationale for ICH with the largest haematoma diameter>2 cm is based on observational data from a blood pressure-lowering trial where such patients were at risk of poor outcome.^24^ Moreover, ICH patients with diameter>2 cm were more likely to receive mannitol in another study.^18^ Visual assessment of maximum haemorrhage diameter has been shown to have strong agreement with measured diameter. It is quick to perform and has been previously shown that the measures are reproducible.^25^ Moreover, visual assessment has the advantage that it can be applied in the absence of special measurement tools in emergency care settings.

The use of consent from a relative, close friend or professional representative is based on large hyperacute stroke trials such as TICH-2 (Tranexamic acid for hyperacute primary IntraCerebral Haemorrhage-2 Trial). This method of consent is essential as the majority of patients will lack capacity. The rationale for 10% mannitol is that it is less likely to crystallise than the previously commonly used 20% infusion and can be stored on a ward without the need for warming cabinets.^26^ The 20% mannitol solution has high osmolality^26^ which could increase the risk of direct venous irritation and skin damage if extravasation occurs. As a result, a large number of UK hospitals, including sites which have expressed interest in this trial, have switched to using mannitol 10% infusion. There is no guidance on which concentration of mannitol is more effective, but an equivalent dose will be administered. The 10% solution is readily available and may have fewer complications. We have chosen 1 g/kg as it has been tested before and not exceeded in previous studies.^27^,^29^ A direct comparison of previous studies shows that for an average patient weighing 70 kg, doses of mannitol varied between 20 and 100 g, and this proposal is well within this range at 70 g/24 hours.^27^,^29^ We anticipate the infusion protocol to be simple and easy to implement. Patients will receive 0.9% saline if the systolic blood pressure reduces to <90 mm Hg. Using 0.9% saline in this setting is not known to worsen cerebral oedema. The trial flowchart has been carefully designed to reflect implementation in UK stroke units. Through this approach, we hope to maximise participant recruitment and clinician engagement. Input was sought from critical care physicians and pharmacists to ensure safety checks, including blood tests, blood pressure monitoring and monitoring of urine output, were incorporated into the trial protocol.

## Supplementary material

10.1136/bmjopen-2025-103776 online supplemental file 1
