# Supplementary material for: Mannitol for cerebral oedema after acute intracerebral haemorrhage (MACE-ICH): protocol for a prospective, randomised, open-label, blinded-endpoint phase IIb trial
Source: BMJ Open. 2025 Jul 28;15(7):e103776. doi: 10.1136/bmjopen-2025-103776 (PMC12306335; doi:10.1136/bmjopen-2025-103776)

Supplementary document

Mannitol for cerebral oedema after acute intracerebral haemorrhage (MACE-ICH): protocol for a prospective, randomised, open-label, blinded-endpoint phase II trial

Neuroimaging analysis

All volumetric analysis of CT scans will be performed by an image analysis group blinded to the treatment allocation. Pseudo-anonymised non-contrast cranial CT scans from baseline and day 5 (+/-2) will be transferred electronically in DICOM format from recruiting sites to the trial coordinating centre using a web-based image transfer portal. Following conversion to NIFTI file format using dcm2niix software (https://github.com/rordenlab/dcm2niix), the intracerebral haemorrhage (ICH), intraventricular haemorrhage (IVH) and oedema will be segmented using the 3D nnU-Net model with Focal loss reported previously by Kok et al (23)to provide ICH, IVH and oedema volumes (**Supplementary Figure**). This model was trained and validated using n=1732 annotated non-contrast CT scans from the TICH-2 trial(24), and achieved concordance correlation coefficients of 0.98, 0.99 and 0.88 for ICH, IVH, and oedema volumes respectively. To provide additional validation of the 3D nnU-Net model, a subset of n=30 scans from MACE-ICH will undergo semi-automated segmentation using ITK-SNAP version 4.2.0 (25) by a radiologist trained in ICH/IVH/oedema segmentation. Comparison of volumes from the AI-based automated and human semi-automated segmentations will be made by intraclass correlation (ICC; type A, absolute agreement; single measures; two-way mixed effects model). This rater has previously demonstrated high ICC for inter-rater (0.95/0.92/0.74) and intra-rater (0.96/0.97/0.97) reliability for ICH/IVH/oedema measurement respectively.

All CT scans will undergo expert radiological review for rating of hydrocephalus and manual measurement of maximal midline shift at the level of the septum pellucidum (**Supplementary Figure**). For this, the DICOM images will be reviewed in the Radiant DICOM viewer (26) which provides a calliper tool for linear measurement. Change in total haematoma volume (ICH +IVH), oedema volume and midline shift at day 5 as well as proportion of patients with ICH expansion, defined as an absolute increase of more than 6 ml will be reported.

Supplementary Figure (1a) Plain CT scan showing hyperdense acute ICH with surrounding oedema and mass effect. (1b) The approximate boundary of the oedema surrounding the ICH is shown by the white arrowheads, and the midline shift is shown as the white double-

headed arrow indicating the shift of the septum pellucidum away from the true midline (white dotted line). (1c) Example of the fully-automated segmentation of ICH (red label) and oedema (green label) using the segmented using the 3D nnU-Net model with Focal loss(23)

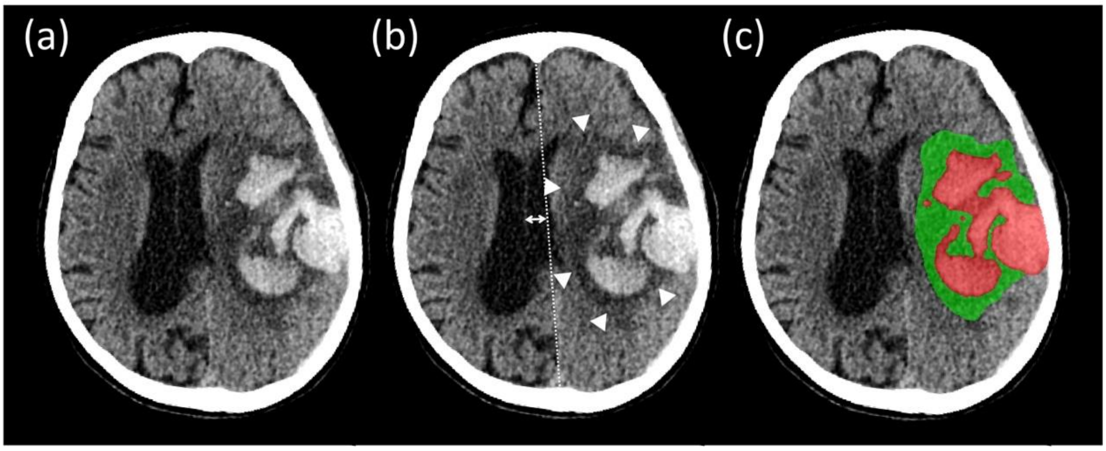

Supplement: online supplemental file 1 [file bmjopen-15-7-s001.pdf]
