# Supplementary material for: Mannitol for cerebral oedema after acute intracerebral haemorrhage (MACE-ICH): protocol for a prospective, randomised, open-label, blinded-endpoint phase IIb trial
Source: BMJ Open. 2025 Jul 28;15(7):e103776. doi: 10.1136/bmjopen-2025-103776 (PMC12306335; doi:10.1136/bmjopen-2025-103776)
Supplement: Supplementary file 2 [file bmjopen-2025-103776.reviewer_comments.pdf]

PEER REVIEW HISTORY

BMJ Open publishes all reviews undertaken for accepted manuscripts. Reviewers are asked to complete a checklist review form and are provided with free text boxes to elaborate on their assessment. These free text comments are reproduced below.

ARTICLE DETAILS

Title (Provisional)

Mannitol for cerebral oedema after acute intracerebral haemorrhage (MACE-ICH): protocol for a prospective, randomised, open-label, blinded-endpoint phase IIb trial

Authors

Krishnan, Kailash; Grace, Emma; Woodhouse, Lisa; Roffe, Christine; Dawson, Jesse; England, Timothy J; Hewson, David W; Dineen, Rob A; Law, Zhe Kang; Pszczolkowski, Stefan; Wells, Keenan; Buck, Amanda; Craig, Jennifer; Havard, Diane; Macleod, Mary Joan; Werring, David J; Doubal, Fergus; Sprigg, Nikola; Bath, Philip

---

VERSION 1 - REVIEW

Reviewer **1**

Name **Arboix, Adrià**

Affiliation **Department of Neurology, Sagrat Cor University Hospital, University of Barcelona**

Date **05-May-2025**

COI **None**

It is unclear whether mannitol is beneficial in the management of cerebral edema after intracerebral hemorrhage (ICH). The authors present a study protocol aimed at assessing the feasibility of a prospective, randomized, open label, multicenter, UK-based, three-arm, blinded-endpoint, phase II trial (MACE-ICH; ISRCTN15383301) of mannitol for patients with acute ICH with, or at risk for, cerebral edema, in order to inform a definitive trial. The authors aim to include 45 ICH participants from 10 UK centers with estimated largest diameter of hematoma volume >2 cm. Participants will be randomized (1:1:1) to 1 g/kg 10% intravenous mannitol as a single-dose, 1 g/kg 10% mannitol followed by a second dose at 24 hours, or standard care alone. MACE-ICH received ethical approval. The authors suggest that the results could have an important impact on daily clinical practice and that mannitol could be rapidly adopted into clinical guidelines if it is shown to reduce death or disability in patients with ICH. The paper is potentially interesting, but some aspects of the manuscript may be improved by considering the following points:

-1.The authors should mention in the text that acute spontaneous lobar cerebral hemorrhages present a different clinical profile and a more severe early prognosis than deep subcortical intracerebral hemorrhages (Biomedicines 2023 Jan 16;11(1):223) and should add that non-hypertensive mechanisms of intracerebral hemorrhages predominate in the lobar location. It would be interesting that the authors propose the presence of differences in their results in relation to hemorrhagic brain topography.

-2. It would be interesting to add in the protocol study the analysis in the subgroup of very old patients (85 years and older) given that demographics, risk factors and outcome are quite different in this age segment of hemorrhagic stroke patients

-3.It would be interesting to add in the text a comment on the relevance of intraventricular extension of acute intracerebral hemorrhage as a determinant of poor outcome (BMC Neurol. 2007 Oct 5;7:32. doi: 10.1186/1471-2377-7-32. PMID: 17919332; PMCID: PMC2169250). - Did the authors consider this in their project?

4.Open lines of research include the efficacy and safety of mannitol in men versus women, or in different etiologic hemorrhagic stroke subgroups, all of which are important and challenging subsets of acute intracerebral hemorrhage that deserve to be evaluated in further studies

---

**Reviewer**	**2**
**Name**	**Latifi, Shahrzad**
**Affiliation**	**Rockefeller Neuroscience Institute, West Virginia University**
**Date**	**03-Jun-2025**
**COI**	**None**

ID bmjopen-2025-103776

This study represents the randomized controlled trial specifically evaluating the feasibility and safety of mannitol administration for cerebral edema in patients with spontaneous intracerebral hemorrhage. It introduces a novel dose-comparative approach by assessing both single and repeated mannitol dosing regimens, addressing an important gap in current clinical practice. Notably, the study incorporates advanced neuroimaging analysis using reproducible volumetric assessment of hemorrhage and edema, enhancing objectivity in outcome

measurement. However, there are several comments that need to be addressed before publication.

Major comments:

1. The study lacks a hypothesis, making its findings on outcome variability and biomarker changes purely exploratory.
2. Enhanced monitoring for adverse events may be necessary for renal dysfunction or electrolyte imbalance.
3. The study lacks biomarker collection and neuroimaging beyond CT, missing the chance to explore mannitol's mechanistic effects beyond volumetric changes.
4. The protocol utilizes a 10% mannitol solution, which differs from the more commonly studied 20% concentration; a citation or justification is needed.

Minor comments:

1. There is a lack of sufficient citations from previous studies(introduction), which may highlight their potential issues.
2. The small sample size (n=45) limits the study to feasibility, with no emphasis on efficacy or safety. Any trends in mortality or disability outcomes are exploratory.
3. The requirement to enroll within 72 hours after onset (and following imaging and consent) may limit recruitment.
4. It is important to ensure that the citation format is consistent throughout.

VERSION 1 - AUTHOR RESPONSE

Reviewer 1 comments:

1. **The authors should mention in the text that acute spontaneous lobar cerebral hemorrhages present a different clinical profile and a more severe early prognosis than deep subcortical intracerebral hemorrhages (Biomedicines 2023 Jan 16;11(1):223) and should add that non-hypertensive mechanisms of intracerebral hemorrhages predominate in the lobar location**

Response: We thank the reviewer for this suggestion. This has now been incorporated in the introduction and referenced.

2. **It would be interesting to add in the protocol study the analysis in the subgroup of very old patients (85 years and older) given that demographics, risk factors and outcome are quite different in this age segment of hemorrhagic stroke patients**

   Response: We thank the reviewer for this suggestion but this is a feasibility trial and not powered to undertake such an analysis.

3. **It would be interesting to add in the text a comment on the relevance of intraventricular extension of acute intracerebral hemorrhage as a determinant of poor outcome (BMC Neurol. 2007 Oct 5;7:32. doi: 10.1186/1471-2377-7-32. PMID: 17919332; PMCID: PMC2169250). - Did the authors consider this in their project?**

   Response: We thank the reviewer for this suggestion. We have included this the relevance of IVH in the introduction and referenced. The inclusion criteria for this trial is broad allowing for patients with intraventricular haemorrhage also to be included.

4. **Open lines of research include the efficacy and safety of mannitol in men versus women, or in different etiologic hemorrhagic stroke subgroups, all of which are important and challenging subsets of acute intracerebral hemorrhage that deserve to be evaluated in further studies.**

   Response: We thank the reviewer for this suggestion. As such, this feasibility trial is not powered to undertake such analysis. We hope to undertake these in a

future, larger trial testing safety as well as efficacy. As such, no change has been made to the manuscript.

Reviewer 2 comments

1. **The study lacks a hypothesis, making its findings on outcome variability and biomarker changes purely exploratory.**

   Response: We thank the reviewer for this comment. We have incorporated a sentence in the Trial design section that the overarching hypothesis is that mannitol will reduce cerebral oedema after acute intracerebral haemorrhage and thereby improve outcomes.

2. **Enhanced monitoring for adverse events may be necessary for renal dysfunction or electrolyte imbalance.**

   Response: We have stated in the manuscript that all adverse events on days 0-1 (including during infusion, Arms 1 and 2 only) and for 24 hours post treatment will be collected. We have clarified in the Outcomes section of the manuscript that during days 1-2 Urea and electrolytes (U&E's); e-GFR; serum osmolality to correlate response to mannitol will be collected.
   In the adverse section of the manuscript, we had highlighted that all SAEs will be collected until day 28, and fatal SAEs and safety outcome events including hyper/hyponatremia and renal impairment will be collected until day 180. The protocol excludes patients with severe renal failure (e-GFR <30 or those on dialysis). As such, we do not think any changes need to be made.

3. **The study lacks biomarker collection and neuroimaging beyond CT, missing the chance to explore mannitol's mechanistic effects beyond volumetric changes.**

   Response: We thank the reviewer for this comment. This trial has been funded and approved to collect the mechanistic effects by volumetric changes using CT scans only and not on biomarkers.

4. **The protocol utilizes a 10% mannitol solution, which differs from the more commonly studied 20% concentration; a citation or justification is needed.**

   Response: We thank the reviewer and wish to highlight in the Discussion section of the manuscript that it was highlighted that the rationale for 10% mannitol is that it is less likely to crystallise than the previously commonly used 20% infusion and can be stored on a ward without the need for warming cabinets. As a result, a large number of UK hospitals including sites which have expressed interest in this trial have switched to using mannitol 10% infusion.

   Following the comment from the reviewer, we have now included a reference for the rationale of using 10% mannitol. We have now added a sentence to clarify that the 20% mannitol solution has high osmolality which could increase the risk of direct venous irritation and skin damage if extravasation occurs.

Minor comments

1. **There is a lack of sufficient citations from previous studies (introduction), which may highlight their potential issues.**

Response: We have now included references in the Introduction section sentence stating ‘Little is known about its effects in ICH: to date studies have been low quality and underpowered.’ We have also included one references each in two earlier sentences: Mannitol is also thought to reduce blood viscosity, facilitating blood flow and oxygen delivery to the brain. As a free-radical scavenger, mannitol might act as a neuroprotectant.

2. **The small sample size (n=45) limits the study to feasibility, with no emphasis on efficacy or safety. Any trends in mortality or disability outcomes are exploratory.**

   Response: This trial was funded and approved to assess feasibility. It is not powered to assess efficacy or safety. As such, analysis on outcomes including mortality or disability would be exploratory.

3. **The requirement to enroll within 72hours after onset (and following imaging and consent) may limit recruitment.**

   Response: We thank the reviewer for the comment and wish to highlight that the rationale for $\leq$ 72 hours was based on when patients are likely to develop brain swelling and/or are at risk of further expansion. This was highlighted and referenced in the Discussion section of the manuscript at the time of the first submission. One key question of this feasibility trial is to examine whether participants could be recruitment within 72 hours of onset and this has been explained. As such, we do not think any changes to be made.

4. **It is important to ensure that the citation format is consistent throughout.**

   Response: We apologise for the error. We have now ensured that the citation format is consistent and aligned with BMJ.

Others:

1. A sentence on when the trial started recruitment of participants and scheduled for closure has now been included in the Methods section.
2. The conclusion section of the manuscript has been removed.
